# Supplementary material for: Plug-and-Play Self-Supervised Denoising for Pulmonary Perfusion MRI
Source: Bioengineering (Basel). 2025 Jul 1;12(7):724. doi: 10.3390/bioengineering12070724 (PMC12292463; doi:10.3390/bioengineering12070724)
Supplement: Supplementary file 1 [file bioengineering-12-00724-s001.zip › Supplementary Information Table S1.pdf]

Supplementary Information Table S1.

Table S1. Demographics Information

	Training		Testing	
	**Adult Patients (n=25)**	**Pediatric Patients (n=4)**	**Adult Patients (n=7)**	**Pediatric Patients (n=1)**
**Male, n (%)**	17	4	7	1
**Age (y)**	52 ± 17.9	9.9 ± 7.8	52.9 ± 22.6	1
**Height (cm)**	172 ± 11.2	141 ± 56.2	172.1 ± 8.1	71.1
**Weight (kg)**	82.3 ± 18.4	65.4 ± 60.4	75.4 ± 14.3	12.2

Eligible patient groups included those with (a) suspected pulmonary embolism (n = 15), (b) hypertension (n = 1), (c) chest pain (n = 6), (d) Marfan syndrome (n = 1), (e) dysphagia (n = 1), (f) Horner's syndrome (n = 1), (g) Anisocoria (n = 1), (h) thoracic outlet syndrome (n = 5), (i) cardiac disease (n = 2), (j) superior vena cava (SVC) syndrome (n = 2) and (k) inflammatory (n = 2). Within the 37 patients, 5 of them were children (8 ± 8 years).
