# Supplementary material for: Plug-and-Play Self-Supervised Denoising for Pulmonary Perfusion MRI
Source: Bioengineering (Basel). 2025 Jul 1;12(7):724. doi: 10.3390/bioengineering12070724 (PMC12292463; doi:10.3390/bioengineering12070724)
Supplement: Supplementary file 1 [file bioengineering-12-00724-s001.zip › Supplementary Information Table S2.pdf]

Supplementary Information Table S2

Table S2: TWIST MRI sequence parameters in training and testing dataset

	**Training (n=29)**	**Testing (n=8)**
**TR (ms)**	2.62 ± 0.31	2.53 ± 0.23
**TE (ms)**	0.94 ± 0.07	0.91 ± 0.03
**Flip angle**	24.2 ± 6.0	26.2 ± 6.5
**Spatial resolution (mm)**	1.25 ± 0.16	1.27 ± 0.13
**Slice thickness (mm)**	1.74 ± 1.11	1.77 ± 1.15
**Bandwidth (Hz)**	717 ± 277	708 ± 284
**Number of slices**	117 ± 36	120 ± 37

3D pulmonary perfusion images were acquired using the time resolved angiography with interleaved stochastic trajectories (TWIST) sequence during free-breathing for 15-17 seconds after contrast injections followed by 10-15 sec breath-hold imaging for high-quality peak perfusion acquisitions then free-breathing for the rest acquisitions. Only the peak perfusion 3D images were selected and sent to the PACS system.
