# Supplementary material for: Plug-and-Play Self-Supervised Denoising for Pulmonary Perfusion MRI
Source: Bioengineering (Basel). 2025 Jul 1;12(7):724. doi: 10.3390/bioengineering12070724 (PMC12292463; doi:10.3390/bioengineering12070724)
Supplement: Supplementary file 1 [file bioengineering-12-00724-s001.zip › Supplementary Information Text S1.pdf]

Supplementary Information Text 1. Full details of AP-BSN and hyperparameters:

The Asymmetric pixel-shuffle Downsampling Blind-Spot Network (AP-BSN) (1) is a self-supervised learning model for image denoising that operates without clean image references. It consists of two main components during training: Pixel Down-sampling (PD=5) and a Blind-Spot Network (BSN). During inference, it includes a Blind-Spot Network (BSN) and a Random Replacing Refinement (R3) process, with no pixel down-sampling applied (PD=1) to increase the sharpness of the output.

AP-BSN loss function (L_{BSN}) is in Equations S1,

$$L_{BSN} = \left\| PD_s^{-1}\left(BSN\big(PD_s(I_n)\big)\right) - I_n \right\|_1 = \|I_{BSN} - I_n\|_1, \quad \text{S1}$$

where I_n is the noisy input, PD_s is PD with stride s, PD_s^{-1} is the inverse PD_s, I_{BSN} is the output after PD_s, BSN and PD_s^{-1}. Random-replacing refinement (R3) is shown in Equations S2,

$$I_{M_i} = M_i \circ I_N + (1 - M_i) \circ I_{BSN}, \quad \text{S2}$$

where M_i is a binary mask with 1 meaning pixel to be replaced, $\circ$ is element-wise matrix multiplication.

PD reduces spatial resolution to enhance denoising capabilities, with a stride factor of 5 during training (PD=5) and 1 during inference (PD=1). The BSN, a dilated network, processes the down-sampled image with 128 base channels and 9 modules.

The network structure starts with an input layer consisting of a convolutional layer with 128 channels followed by ReLU, then splits into two branches. The first branch begins with a central masked 3×3 convolution layer (stride=2), while the second branch begins with a central masked 5×5 convolution layer (stride=3) as shown in Figure 1B. Each branch then applies a 1×1 convolution layer followed by a ReLU activation.

In Figure 1B, each branch applies 9 Dilated Convolution Blocks. This is followed by a final 1×1 convolution layer and a ReLU activation in each branch. The outputs of both branches are then concatenated and passed through a refinement block containing four sequential 1×1 convolution layers, each followed by a ReLU activation, to produce the final denoised image.

In Figure 1C, random Replacing Refinement (R3) process during inference to enhance the denoising performance: R3 enhances denoising during inference: it generates 16 random masks with a 16% replacement probability, replacing parts of the denoised output with

corresponding noisy input pixels. These modified inputs are processed independently through the network, and the outputs are averaged to produce the result.

Training hyperparameters: batch size 20, max epoch 20, learning rate initialized at 1e-4 with a step scheduler (step size of 8, gamma of 0.1), optimizer Adam with beta values [0.9, 0.999], loss function L1 loss (Eq. S1), 200 warmup iterations to stabilize training.
