# Supplementary material for: Plug-and-Play Self-Supervised Denoising for Pulmonary Perfusion MRI
Source: Bioengineering (Basel). 2025 Jul 1;12(7):724. doi: 10.3390/bioengineering12070724 (PMC12292463; doi:10.3390/bioengineering12070724)
Supplement: Supplementary file 1 [file bioengineering-12-00724-s001.zip › Supplementary Information Text S2.pdf]

Supplementary Information Text 2. Full details of PnP-BSN using the alternating direction method of multipliers (ADMM):

$$arg \min_{x} \frac{1}{2} ||\boldsymbol{y} - \boldsymbol{x}||^2 + \lambda g(\boldsymbol{x})$$

where $\boldsymbol{y}$ is the original noisy image, $\boldsymbol{x}$ is the denoised image, λ is the weighting parameter to balance the data fidelity and the image prior using AP-BSN, $g(\boldsymbol{x})$ is a denoiser.

If $g(\boldsymbol{x})$ is implicitly modeled by the denoiser $\boldsymbol{z} = \mathcal{D}_\sigma(\boldsymbol{x} + \boldsymbol{u})$, here a trained AP-BSN ($\mathcal{D}_\sigma$), then,

$$\mathcal{L}(\boldsymbol{x}, \boldsymbol{z}, \boldsymbol{u}) = \frac{1}{2} ||\boldsymbol{y} - \boldsymbol{x}||^2 + \boldsymbol{u}(\boldsymbol{x} - \boldsymbol{z}) + \frac{\rho}{2} ||\boldsymbol{x} - \boldsymbol{z}||^2$$

where $\mathcal{L}(\boldsymbol{x}, \boldsymbol{z}, \boldsymbol{u})$ is an augmented Lagrangian for the optimization problem, $\boldsymbol{z}$ is an auxiliary variable, the first term enforces the denoised image $\boldsymbol{x}$ to be close to the noisy image $\boldsymbol{y}$, the second term is a regularization term that enforces the output to be close to the auxiliary variable $\boldsymbol{z}$, and $\boldsymbol{u}$ is a dual variable. ρ is selected as 1 to balance the noise control and image fidelity.

In PnP-BSN, the AP-BSN is used as the denoiser within the framework, $\boldsymbol{x}$ is solved using ADMM method,

$$arg \min_{x,z} \frac{1}{2} ||\boldsymbol{y} - \boldsymbol{x}||^2 + \frac{\rho}{2} ||\boldsymbol{x} - \boldsymbol{z}||^2, \quad \text{subject to } \boldsymbol{z} = \mathcal{D}_\sigma(\boldsymbol{x} + \boldsymbol{u}).$$

Here is a detailed explanation of the algorithm and its steps:

Initialization:

$\boldsymbol{x}$: Initially set to the noisy image $\boldsymbol{y}$.

$\boldsymbol{z}$: Initially set to the denoised output of $\boldsymbol{x}$ using AP-BSN denoiser trained using the pulmonary perfusion MRI images.

$\boldsymbol{u}$: Initially set to a zero matrix of the same shape as $\boldsymbol{y}$.

ρ: set to 1 to balance the original noisy image and the denoised image.

Using a maximum of $k = 3$ iterations here is justified because AP-BSN is highly effective in denoising.

Update step for the $\boldsymbol{k^{th}}$ iteration:

$\boldsymbol{x}$ is updated using the formula:

$$\boldsymbol{x}^{(k+1)} = \arg\min_{x} \frac{1}{2} ||\boldsymbol{y} - \boldsymbol{x}||^2 + \frac{\rho}{2} ||\boldsymbol{x} - \boldsymbol{z}^{(k)} + \boldsymbol{u}^{(k)}||^2$$

$$\boldsymbol{x}^{(k+1)} = \frac{\boldsymbol{y} + \rho\left(\boldsymbol{z}^{(k)} - \boldsymbol{u}^{(k)}\right)}{1 + \rho}$$

Update $\boldsymbol{z}$:<br>$$\boldsymbol{z}^{(k+1)} = \mathcal{D}_\sigma\left(\boldsymbol{x}^{(k+1)} + \boldsymbol{u}^{(k)}\right)$$<br>Update $\boldsymbol{u}$:<br>$$\boldsymbol{u}^{(k+1)} = \boldsymbol{u}^{(k)} + \boldsymbol{x}^{(k+1)} - \boldsymbol{z}^{(k+1)}$$
The output image achieves a balanced improvement in quality, reducing noise while maintaining image fidelity and details of pulmonary perfusion MRI.
