# Supplementary material for: Plug-and-Play Self-Supervised Denoising for Pulmonary Perfusion MRI
Source: Bioengineering (Basel). 2025 Jul 1;12(7):724. doi: 10.3390/bioengineering12070724 (PMC12292463; doi:10.3390/bioengineering12070724)
Supplement: Supplementary file 1 [file bioengineering-12-00724-s001.zip › Supplementary Information Text S3.pdf]

Supplementary Information Text 3. Full details of fractal analysis and fractal dimension:

The local fractal analysis algorithm implemented in the proposed method is based on the blanket method (1,2), which iteratively adjusts a top and bottom surface blanket over the image texture. For each pixel, the direct neighbors in a fixed 3×3 window are checked. The area of the blanket is calculated at each iteration, and a plot of the logarithm of the area against the logarithm of the iteration step yields a line with a negative slope is used to compute the fractal dimension (FD) as 2 minus the slope. The algorithm uses 44 iterations, determined to be optimal for capturing the fractal characteristics of the texture. The FD reflects the geometrical complexity of the image by quantifying the texture complexity in a spatially two-dimensional grayscale image. FD values range from 2 to 3, with a value approaching 2.0 indicating a flatter, less complex texture, and higher value indicates higher complexity. In this work, the FD is a metric for assessing the pulmonary perfusion image complexity, which helps in identifying regions with varying degrees of vascular and textural complexity. Higher FD values typically indicate more complex and heterogeneous regions such as the edge of the vessels, which is crucial for quantifying the perfusion patterns.

Fractal analysis visualization: For visualizations, pseudo-color maps (e.g., ‘hot’ or ‘jet’) were applied solely for illustrative purposes. The chosen contrast settings and color maps were selected to emphasize regions of contrast enhancement, vascular structure, and perfusion heterogeneity in the lung region. These settings were fixed across all methods to ensure unbiased visual comparison of image quality and noise suppression.
