# Supplementary material for: Plug-and-Play Self-Supervised Denoising for Pulmonary Perfusion MRI
Source: Bioengineering (Basel). 2025 Jul 1;12(7):724. doi: 10.3390/bioengineering12070724 (PMC12292463; doi:10.3390/bioengineering12070724)
Supplement: Supplementary file 1 [file bioengineering-12-00724-s001.zip › Supplementary Information Text S4.pdf]

Supplementary Information Text 4. Full details of radiologist scoring criteria:

Three image quality criteria were assessed for each denoising method: signal-to-noise ratio (SNR), sharpness, and overall image quality. These were evaluated subjectively by two radiologists using a standardized 5-point Likert scale (1 being the worst and 5 being the best).

• SNR was defined as the radiologist's perception of noise in the image, where a higher score indicates lower visible noise.

• Sharpness was defined as the perceived clarity of anatomical structures and the degree to which structural blurring was suppressed by the denoising algorithm.

• Overall image quality reflected a combined impression of noise, structural fidelity, and sharpness, using the original noisy image as a reference.

Scoring was performed using the following 5-point scale:

(1) Poor: high noise and/or significant blurring;

(2) Fair: reduced noise and/or significant blurring;

(3) Good: low noise but moderate blurring;

(4) Very good: low noise and well-controlled blurring;

(5) Excellent: no significant noise or blurring.

Radiologists viewed one PowerPoint slide per patient, which displayed all the slices of denoised images from the same subject but from three different methods (Methods 1–3) presented in random order from left to right. The original noisy image was also shown as a reference. All image denoising and evaluation were performed on grayscale images. For visualization, a consistent linear grayscale window was applied to preserve relative intensity differences and enable visual comparison. Each method was scored independently in all three metrics, and the scores were recorded using a structured Excel sheet for later statistical analysis.
