# Supplementary figures and images for: Plug-and-Play Self-Supervised Denoising for Pulmonary Perfusion MRI
Source: Bioengineering (Basel). 2025 Jul 1;12(7):724. doi: 10.3390/bioengineering12070724 (PMC12292463; doi:10.3390/bioengineering12070724)

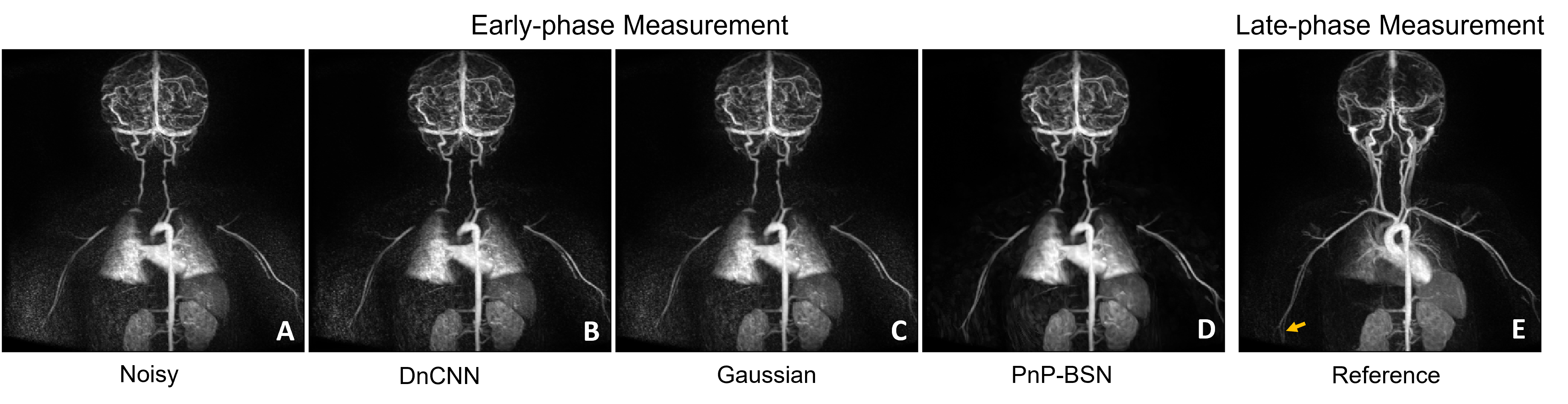

Supplement: Supplementary file 1 [file bioengineering-12-00724-s001.zip › figure S1.png]
